# Effects of Exercise on Gut Microbiome and Serum Metabolomics in Post-Traumatic Osteoarthritis Rats

**DOI:** 10.3390/metabo15050341

**Published:** 2025-05-20

**Authors:** Xiaoxia Hao, Xingru Shang, Yiwen Zhang, Wenjie Hou, Ruimin Chi, Chunran Pan, Jiawei Liu, Xiaofeng Deng, Jiaming Zhang, Tao Xu

**Affiliations:** 1 Department of Rehabilitation, Tongji Hospital, Tongji Medical College, Huazhong University of Science and Technology, Wuhan 430030, China; haoxiaoxia@hust.edu.cn (X.H.); 205156@hospital.cqmu.edu.cn (X.S.); m202376519@hust.edu.cn (Y.Z.); m202276333@hust.edu.cn (W.H.); d202282094@hust.edu.cn (R.C.); d202482373@hust.edu.cn (C.P.); ljw20tj@hust.edu.cn (J.L.); d202382160@hust.edu.cn (X.D.); 2 Department of Rehabilitation, The First Affiliated Hospital of Chongqing Medical University, Chongqing 400015, China; 3 Clinical Innovation & Research Center (CIRC), Shenzhen Hospital, Southern Medical University, Shenzhen 518100, China

**Keywords:** osteoarthritis, exercise, gut–joint axis, metabonomics

## Abstract

**Objective:** The aim of this work is to investigate the impact of exercise on gut microbiome composition, serum metabolites, and their correlation with osteoarthritis (OA) severity. **Methods:** Thirty-six Sprague-Dawley (SD) rats were randomly divided into four groups: Sham rats without treadmill walking (Sham/Sed group, *n* = 9), Sham rats with treadmill walking 2 months (Sham/TW2M group, *n* = 9), PTOA rats without treadmill walking (PTOA/Sed group, *n* = 9), and PTOA rats with treadmill walking 2 months (PTOA/TW2M group, *n* = 9). The PTOA model was induced by transection of the anterior cruciate ligament (ACLT) and destabilization of the medial meniscus (DMM). Histological evaluation and micro-CT analysis were performed to observe the pathological changes in cartilage and subchondral bone, respectively. Additionally, we conducted 16S rDNA sequencing of fecal samples and untargeted metabolomic analysis using liquid chromatography–mass spectrometry (LC–MS) of serum samples to detect the alteration of gut microbiota composition and metabolites. **Results:** Exercise effectively mitigated OA-related pathological changes, including articular cartilage degeneration and subchondral bone loss. Moreover, 16S rDNA sequencing analysis of gut microbiome revealed a decreased abundance of *Bacteroidetes* (*p* < 0.01), *Bacteroidia* (*p* < 0.01), *Rikenellaceae* (*p* < 0.01), [*Paraprevotellaceae*] (*p* < 0.01), and *Paraprevotella* (*p* < 0.01) but an increase in *Firmicutes* (*p* < 0.01) in PTOA/TW2M group rats compared with PTOA/Sed group as a response to exercise. In addition, the results of metabolomics analysis showed that exercise treatment contributed to the upregulation of Daidzein and Anthranilic acid and downregulation of 1-Palmitoyllysophosphatidylcholine. Moreover, the correlation analysis showed that *Rikenellaceae* significantly positively correlated with both OARSI (r = 0.81, *p* < 0.01) and Mankin score (r = 0.83, *p* < 0.01) and negatively correlated with the serum level of Anthranilic acid (r = −0.56, *p* < 0.01) and Daidzein (r = −0.46, *p* < 0.01). **Conclusions:** Exercise can effectively mitigate OA through slowing down articular cartilage degeneration and subchondral bone loss, modulating gut microbiota composition, and increasing beneficial metabolites.

## 1. Introduction

Osteoarthritis (OA) is a prevalent joint disorder characterized by extensive cartilage degradation, subchondral bone sclerosis, osteophyte development, and alterations in various other joint components [1]. As estimated, OA cases increased from 247.51 million in 1990 to 527.81 million in 2019 due to population growth and aging, resulting in a substantial economic burden [2]. It represents the primary cause of global disability among the aging population, leading to joint pain, stiffness, swelling, restricted mobility, physical dysfunction, and diminished quality of life [3]. It is now widely recognized that low-grade chronic inflammation, associated with various OA-relevant risk factors including age, diet, trauma, and obesity, is crucial in the advancement of OA [4]. Post-traumatic osteoarthritis (PTOA) is one phenotype of OA, triggered by joint injury. DMM and ACLT surgical models, either independently or together, are efficient and consistent techniques to induce OA, which can imitate the human pathogenesis of post-traumatic phenotypes [5]. It has been suggested that perpetuating an inflammatory response to joint trauma plays an important role in the onset and progression of PTOA [6]. One possible driver of this inflammation, as observed in a range of other chronic conditions, is disruption of the gut microbiome [7].

The gut microbiome, comprising approximately 35,000 bacterial species, colonizes the host’s intestinal tract, of which the most dominant phyla are Firmicutes and *Bacteroidetes* [8]. Gut microbiome impacts the host immune system and regulates metabolic balance through microbial metabolites and bacterial structural components [9]. Gut microbiome dysbiosis denotes an imbalance in the composition and function of intestinal microbes, resulting in dysregulation of the metabolism and disturbance of inflammation and immune reactions [10]. This review summarizes relevant animal and human studies relating to gut microbiome and OA to explore the potential gut–joint axis in OA [11]. Ulici et al. reported that specific pathogen-free (SPF) mice exhibited higher articular cartilage structure scores compared to germ-free (GF) mice following DMM surgery, which suggests the contribution of gut microbiome to the development of injury-induced OA [12]. Guan et al. showed that antibiotic-induced gut microbiota dysbiosis could reduce the serum level of lipopolysaccharide (LPS) and suppress the inflammatory response to alleviate the progression of OA mice induced by DMM surgery [13]. In addition, Boer et al. indicated that the abundance of Streptococcus positively correlated with knee pain in patients with OA based on the largest cohort study [14]. Together, these findings support the fact that there is a strong relationship between gut microbiome and OA pathogenesis. Specifically, dysbiosis of the gut microbiome triggered increased systemic and local levels of microbe-associated pro-inflammatory metabolites, including LPS, peptidoglycan (PGN), and free fatty acids (FFAs) [15]. These key molecules transmitted signals from the gut to systemic tissues, leading to low-grade inflammation, metabolic endotoxemia, macrophage infiltration, and joint damage, which mechanistically contributed to the etiopathology of OA [16].

Currently, with the development of metabolomics technology, focusing on altered metabolites induced by variations in gut microbiome offers a fresh perspective for understanding and treating OA [17]. Rushing et al. found that OA subjects had significant disruptions in microbial metabolites such as propionic acid, indoles, and other tryptophan metabolites [18]. Therefore, manipulating the metabolic activity of gut microbiota may provide a potential approach for OA management. This strategy may include dietary supplementation with probiotics or prebiotics, fecal microbiota transplantation, and exercise [19,20,21]. Recent studies showed that moderate treadmill-walking exercise can notably mitigate joint damage and inflammation in OA rats [22]. However, the specific mechanism remains elusive and needs to be further explored. Furthermore, the impact of exercise on gut microbiome compositions and serum metabolites in the pathogenesis of OA necessitates comprehensive elucidation.

Based on this, we hypothesize that exercise may suppress articular cartilage damage and subchondral bone loss, with this effect possibly being mediated by the manipulation of gut microbiome and corresponding metabolites. To investigate this, we conducted 16S rRNA sequencing on fecal samples and untargeted metabolomics analysis on serum samples collected from rats in each group. The objective was to evaluate the impact of exercise on gut microbiome composition of PTOA rats induced by DMM + ACLT surgery, along with the abundance of serum metabolites, and their correlation with OA severity. These results may provide a theoretical foundation for understanding the therapeutic effects of exercise on OA and support its wider application.

## 2. Materials and Methods

### 2.1. Animals

Thirty-six 9-week-old male SD rats weighing 180–250 g were obtained from the Animal Center of Tongji Hospital. All animal experiments complied with current ethical regulations and were approved by the Committee on the Ethics of Animal Experiments of Tongji Hospital, Tongji Medical College (permission number: TJH-202007010). We adhered to the ARRIVE guidelines [23] and included the ARRIVE checklist as Supplementary Materials. The SD rats were individually housed in per standard ventilated facility filled with irradiated sawdust at constant temperature (22 ± 1 °C), humidity (45%), and a regular 12 h light/dark cycle. All animals could move freely in the cages and had unrestricted access to sterilized food and water.

### 2.2. Study Design and Exercise Protocols

Before the start of the experiment, all animals were randomly assigned to four groups: Sham rats without treadmill walking (namely, Sham/Sed group, *n* = 9), Sham rats with treadmill walking 2 months (namely, Sham/TW2M group, *n* = 9), PTOA rats without treadmill walking (namely, PTOA/Sed group, *n* = 9), and PTOA rats with treadmill walking 2 months (namely, PTOA/TW2M group, *n* = 9). PTOA group rats underwent surgical procedures after anesthesia to induce OA models of ACLT and DMM as previously described [21].

**Figure 1:**
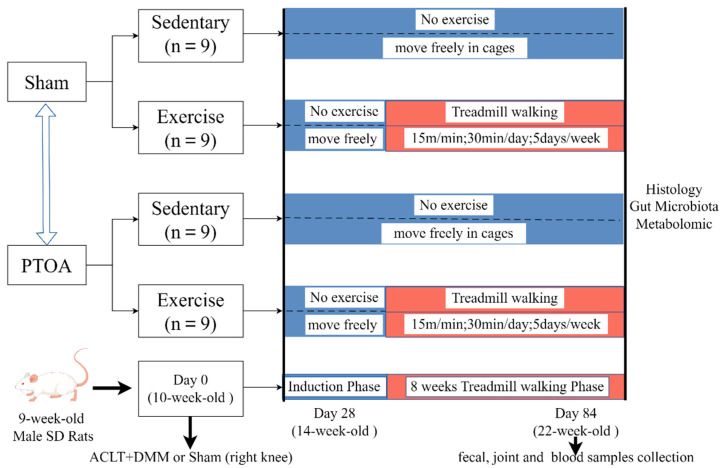
Schematic diagram showing procedures used in the animal model experiment.

The sample size for per group was determined using the Proc Power procedure in SAS 21 (version 9.4) with a significance level (α) at 0.05 and a power of 80%. A minimum sample size of five rats per group is based on prior publication [24] to detect a pre-specified effect size. Therefore, our sample size met the requirement and was sufficient to observe meaningful distinctions in the histological score of the joint. Exercise training was performed at 4 weeks after joint surgery on a flatbed treadmill at a rate of 15 m/min for 30 min/d, 5 days/week. The body weight of rats was measured once per week. The animals were euthanized at 22 weeks of age. Prior to euthanasia, fresh fecal samples and blood samples were collected for analysis. The protocols employed in this study are illustrated in Figure 1.

### 2.3. Micro-Computed Tomography (Micro-CT) Analysis of Subchondral Bone

Following euthanasia, the knee joints of all rats were scanned using a micro-CT system (micro-CT 50 Scanco Medical, Bassersdorf, Switzerland) with 10.5 µm voxel size, 100 kV voltage, and 98 µA current. Reconstructed images and data were processed with three-dimensional built-in software. We analyzed parameters including bone volume/tissue volume fraction (BV/TV), trabecular thickness (Tb.Th), trabecular number (Tb.N), and trabecular separation (Tb.Sp) as previously described [25].

### 2.4. Histological Analysis of Articular Cartilage

Following micro-CT scanning, knee joints were fixed in a 4% paraformaldehyde solution for 48 h at room temperature, decalcified in 10% EDTA solution at 4 °C for 4 weeks, and dehydrated with graded ethanol solutions. The joints were embedded in paraffin in the sagittal plane, and 10 to 20 slides with 4 µm sections were prepared for histological assessments. Subsequently, articular cartilage degeneration of the medial tibial plateau (MTP) was scored by the Osteoarthritis Research Society International (OARSI) scoring system (0–6) [26] and modified Mankin scoring system (0–14) [27], which involve evaluation of chondrocyte loss, articular cartilage structure change, and matrix fibrillation/loss. Three independent, experienced researchers will perform scoring in a blinded way and calculate the mean value for each slide. The mean score of ten randomly selected slides was computed to represent a single sample.

### 2.5. DNA Extraction, 16S rRNA Amplification, and Illumina Miseq Sequencing

Total bacterial genomic DNA was extracted from each rat fecal sample (200 mg) using the Fast DNA SPIN extraction kits (MP Biomedicals, Santa Ana, CA, USA) in accordance with the manufacturer’s instructions. The concentration of extracted DNAs was determined by a NanoDrop ND-1000 spectrophotometer (Thermo Fisher Scientific, Waltham, MA, USA). PCR amplification targeted the V3–V4 region of the bacterial 16S rRNA genes with universal primers [28]. PCR products were purified by Agencourt AMPure Beads (Beckman Coulter, Brea, CA, USA) and quantified using the PicoGreen dsDNA Assay Kit (Invitrogen, Waltham, CA, USA). Quantitative PCR products were prepared for sequencing on Illumina MiSeq by Shanghai Personal Biotechnology Co., Ltd. (Shanghai, China).

### 2.6. Bioinformatics Analysis of Sequencing Data

The optimized sequences were obtained through quality control, filtering, and splicing of raw reads. Sequences with  ≥97% similarity were grouped into the same operational taxonomic units (OTUs) by Usearch version 2.3.4. The dereplication was performed using DADA2 to obtain feature sequences. Then, the taxonomic classification and annotation of OTUs were executed using Quantitative Insights into Microbial Ecology 2 (QIIME2) software and the Greengenes Database [29]. Bacterial taxonomy was determined using relative abundance. A Venn diagram was created to compare the OTUs of the groups. The alpha diversity indices (observed OTUs, Chao1, Shannon, and Simpson) were calculated by QIIME2. Beta diversity was performed based on Weighted UniFrac distance using mothur. The comparison of relative abundances of bacterial taxa of different groups was conducted using the R package (version 3.5.3).

### 2.7. Untargeted Metabolomic Profiling of Serum Samples

Approximately 100 μL of serum was thawed at 4 °C and precipitated with 400 µL ice-cold methanol/acetonitrile (1:1, *v*/*v*; including 10 μL DL-o-Chlorophenyl alanine (2.9 mg/mL) as internal standard); 200 µL supernatants were transferred into a new tube and evaporated to dryness after vortexing and centrifugation. To evaluate the stability and repeatability of the liquid chromatograph mass spectrometer (LC–MS) system, quality control (QC) samples were created by combining 10 μL from each sample. The metabolomics profiling was conducted with ultra-high-performance liquid chromatography (1290 Infinity LC, Agilent Technologies, Santa Clara, CA, USA) coupled with a quadrupole time-of-flight system (AB Sciex TripleTOF 6600, AB SCIEX, Framingham, MA, USA) at Shanghai Applied Protein Technology Co., Ltd. (Shanghai, China). [30]. The separation of prepared samples was injected on an ACQUIY UPLC BEH column (2.1 × 100 mm, 1.7 µm, Waters, Milford, MA, USA) with a flow rate of 0.5 mL/min at 25 °C. The mobile phase was made up of water containing 25 mM ammonium acetate and 25 mM ammonium hydroxide (A) and acetonitrile (B). The gradient elution was performed as reported by Zhang et al. [31]. After separation, mass data acquisition was carried out on an AB Triple TOF 6600 Mass Spectrometer (AB SCIEX, Framingham, MA, USA) equipped with a dual electrospray ionization source (ESI) operating in positive and negative ion modes. The ESI source parameters were set in accordance with the description by Zhang et al. [31]. In the mode of MS acquisition, TOF MS scan time was set at 0.20 s/spectra, and the data were acquired over the *m*/*z* range 60–1000 Da. In the mode of auto MS/MS acquisition, product ion scan accumulation time was 0.05 s/spectra, and the data were collected from 25 to 1000 *m*/*z*. The detailed parameters were outlined by Zhang et al. [31].

### 2.8. Statistical Analysis of Metabolite Profiles

The raw mass spectrometry data (wiff.scan files) were converted to MzXML format by Proteo Wizard software (version 3.0.9134), and then, the nonlinear alignment, automatic integration, and extraction were carried out within the XCMS program (version 3.5.1). Metabolites were identified by comparing their mass spectra with an established in-house database according to available authentic standards. After normalizing total peak intensity, the data were analyzed using SIMCA-P (version 14.1) for multivariate analysis, including orthogonal partial least-squares discriminant analysis (OPLS-DA). The robustness of the OPLS-DA model was assessed by sevenfold cross-validation and response permutation testing. The variable importance for projection (VIP) value from the OPLS-DA model was calculated to show its significance in classification. With VIP value > 1 and *p* values < 0.05, candidate metabolites were regarded as potential biomarkers [32].

### 2.9. Statistical Analysis

All experiments were repeated independently three times, with data presented as means  ±  standard deviation (SD). For parametric tests, data comparisons among multiple groups were conducted using one-way analysis of variance (one ANOVA) followed by Tukey’s post hoc test, while comparisons between two groups were conducted using Student’s *t*-test. Furthermore, the Kruskal–Wallis H test was performed to analyze the nonparametric data (histological scores). GraphPad Prism software version 7.0 was utilized for data analysis and graph production. *p*  <  0.05 was considered statistically significant. In the R software platform (version 3.5.3), the stats package was applied to acquire Spearman’s correlation coefficient to analyze the correlation between some significantly changed metabolites, gut microbiome, and relevant physiological parameters of OA. The correlation threshold was chosen as *p* < 0.05.

## 3. Results

### 3.1. Exercise Attenuates PTOA-Relevant Phenotypes of Cartilage–Subchondral Bone Unit

**Figure 2:**
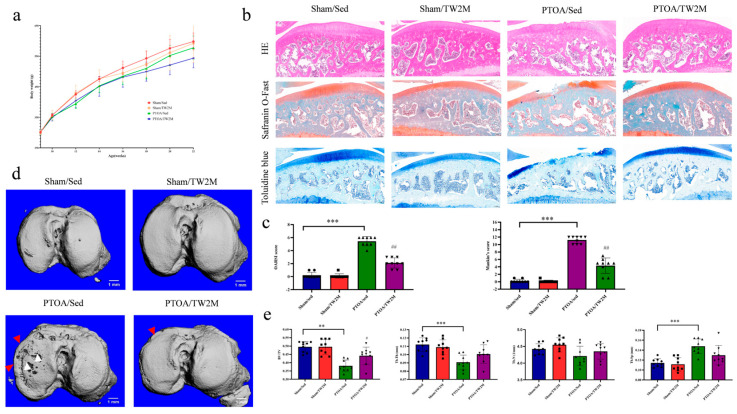
Exercise attenuates PTOA-relevant phenotypes of cartilage–subchondral bone unit. (**a**) The body weight of rats. (**b**,**c**) The representative images of H&E, Safranin O-Fast, and Toluidine blue staining (scale bar = 200 μm, black) in a sagittal plane, OARSI, and modified Mankin score of the Sham/Sed, Sham/TW2M, PTOA/Sed, and PTOA/TW2M groups. (**d**) Micro-CT representative images of the tibial subchondral bone of each group (scale bar = 1 mm, white). Subchondral perforations (white arrow head) and osteophyte mineralization (red arrow head) were confirmed. (**e**) The histograms represent the parameters of tibial trabecular bone, trabecular number (Tb.N), volume/tissue volume (BV/TV), trabecular thickness (Tb.Th), and trabecular separation (Tb.Sp). Data are presented as the mean ± SD, *n* = 9. ** *p* < 0.01, *** *p* < 0.001, PTOA/Sed versus Sham/Sed, one-way ANOVA; # *p* < 0.05, ## *p* < 0.01, PTOA/Sed versus PTOA/TW2M, one-way ANOVA.

Rats’ body weights were recorded weekly to exclude the factor of obesity and overloading on OA severity. As shown in Figure 2a, at the start of the experiment, body weight was similar among the four groups. Throughout the experiment, body weight increased significantly across all groups, with no notable differences at the end of the experiment.

We used H&E, Safranin O-Fast, and Toluidine blue histological staining to evaluate articular cartilage degeneration. As shown in Figure 2b, the cartilage degeneration observed in the PTOA/Sed group was characterized by vertical fissures extending to the deep zone, decreased chondrocytes, and deficiency in cartilage matrix staining, which were consistent with higher OARSI score and modified Mankin score compared with Sham/Sed group (Figure 2c). Micro-CT observations revealed subchondral bone lesions in the PTOA/Sed group, characterized by bone loss, subchondral perforations, and osteophyte mineralization (Figure 2d). Moreover, the parameters of subchondral trabecular bone, including BV/TV and Tb.Th, were significantly lower than the Sham/Sed group (Figure 2e). These alterations highlight the disrupted integrity of the cartilage–subchondral bone unit in the PTOA animal model. However, the cartilage degeneration in the PTOA/TW2M group was mitigated as a response to 8-week treadmill walking compared to the PTOA/Sed group (Figure 2b,c). Simultaneously, the feedback of subchondral bone characterized by the reduced bone loss and higher BV/TV was also detected in the exercised animals (Figure 2d,e). Collectively, these findings validate the hypothesis that exercise prevents the progression of PTOA-relevant cartilage degeneration and subchondral bone loss.

### 3.2. Exercise Modifies the PTOA-Relevant Gut Dysbiosis

**Figure 3:**
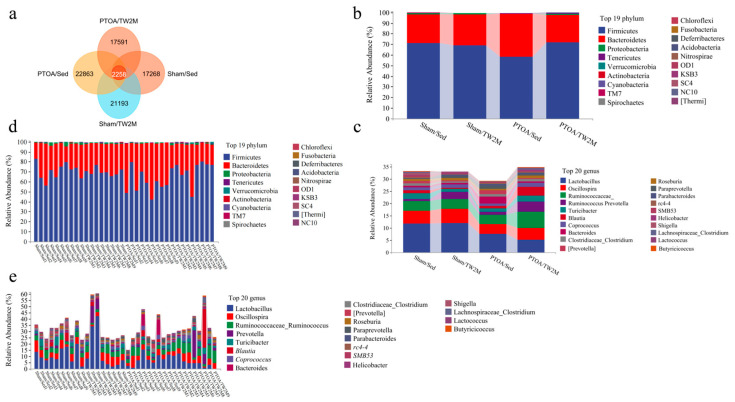
The composition of the gut microbiome in the exercised PTOA animals. (**a**) A Venn diagram was generated to compare OTUs among four groups and to describe OTUs that were specific to the four groups. (**b**–**e**) Taxonomic profiles of the fecal bacteria through 16S rRNA gene sequencing show the relative abundance of the top 19 phyla (**b**,**d**) and the top 20 genera (**c**,**e**) from four groups.

To reveal the influence of joint injury and treadmill walking on gut microbiome, 16S rRNA gene sequencing was performed targeting on V3–V4 region of 36 fecal samples from Sham/Sed, Sham/TW2M, PTOA/Sed, and PTOA/TW2M groups to assess the microbiome composition and diversity. Based on 97% sequence similarity, we identified 78,915 OTUs; 2258 of which existed across all groups were consequently designated as core OTUs. The core OTUs accounted for 2.86% of all OTUs, whereas 17,268, 21,193, 22,863, and 17,591 OTUs were uniquely identified in the Sham/Sed, Sham/TW2M, PTOA/Sed, and PTOA/TW2M groups, respectively (Figure 3a). The OTUs were categorized into 19 bacterial phyla, 33 classes, 78 orders, 141 families, 290 genera, and 407 species. Based on the sequence read counts, the top 19 phyla (Figure 3b,d) and top 20 genera (Figure 3c,e) in relative abundance of the fecal microbiome are presented. The most dominant phyla were *Firmicutes* and *Bacteroidetes* across all four groups, followed by Proteobacteria and Tenericutes, which collectively comprised 99.50%, 99.26%, 99.22%, and 99.09% of the reads in Sham/Sed, Sham/TW2M, PTOA/Sed, and PTOA/TW2M, respectively. At the genus level, Lactobacillus was the most prevalent in the four groups, followed by Oscillospira, Ruminococcus, Prevotella, Turicibacter, and Blautia. These six genera accounted for 25.35%, 26.41%, 18.97%, and 26.70% of the sequences of the Sham/Sed, Sham/TW2M, PTOA/Sed, and PTOA/TW2M groups.

**Figure 4:**
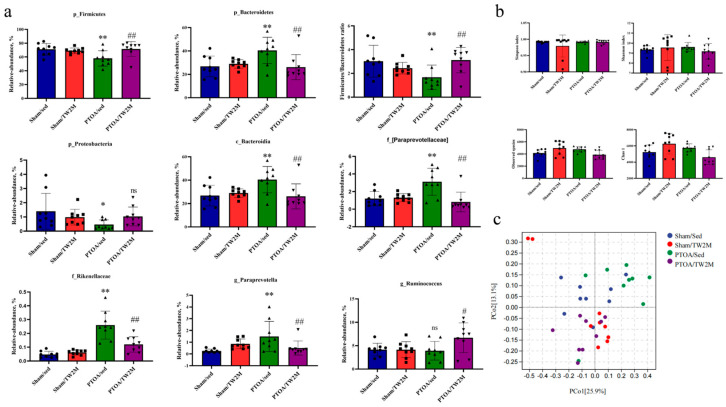
The altered abundance and diversity of the gut microbiome in the exercised PTOA animals. (**a**) Comparison of the phylum, class, family, and genus of fecal gut microbiome composition of four groups. (**b**) Comparison of alpha diversity including Shannon index, Simpson index, Chao1, and Observed species. (**c**) Principal coordinate analysis based on Weighted UniFrac distance. Data are presented as the mean ± SD, *n* = 9. * *p* < 0.05, ** *p* < 0.01, PTOA/Sed versus Sham/Sed, one-way ANOVA; # *p* < 0.05, ## *p* < 0.01, PTOA/Sed versus PTOA/TW2M, one-way ANOVA; ns: not significant.

Differences of fecal microbiome at the phylum, class, family, and genus levels were identified among four groups (Figure 4a). At the phylum level, compared with Sham/Sed group rats, the relative abundance of Firmicutes and Proteobacteria was markedly decreased, and the relative abundance of *Bacteroidetes* was markedly increased in PTOA/Sed group, as well as the decreased *Firmicutes/Bacteroidetes* ratio, indicating PTOA-relevant gut dysbiosis. However, PTOA/TW2M group rats exhibited significantly increased *Firmicutes* and *Firmicutes/Bacteroidetes* ratio but significantly decreased *Bacteroidetes* compared with PTOA/Sed group rats as a response to exercise. At the class level, the relative abundance of *Bacteroidia* was increased in the PTOA/Sed group compared to the Sham/Sed group, while the increased alterations were attenuated in the PTOA/TW2M group. Moreover, the relative abundances of the family [*Paraprevotellaceae*] and *Rikenellaceae* were the highest in the PTOA/Sed group, while a remarkable decrease was observed in the PTOA/TW2M group. At the genus level, PTOA/Sed group presented a higher abundance of *Paraprevotella* compared to the Sham/Sed group rats. Interestingly, *Paraprevotella* remarkably decreased in the PTOA/TW2M group. Additionally, genus *Ruminococcus* was highly enriched in the PTOA/TW2M group. In light of the significant alterations in the composition of the rats’ gut microbiome due to experimental injury and exercise, we evaluated the alpha and beta diversity of the fecal microbiota. The Sham/TW2M group had the highest Chao1 and Observed species, with significant differences in the Shannon and Simpson indices across the four groups (Figure 4b). Through principal coordinate analysis (PCoA) based on Weighted UniFrac distance, we found that the gut microbiota of rats was less dispersed in the Sham/Sed, PTOA/Sed, and PTOA/TW2M groups, but there was obvious segregation in the Sham/TW2M group (Figure 4c). Collectively, these findings indicate that joint injury and exercise both alter the composition and diversity of the intestinal microbiome. Moreover, the microbiome may act as a possible connection between OA and exercise, attributable to the ameliorative effects of exercise on OA-associated microbial alterations.

### 3.3. Exercise Modifies the PTOA-Relevant Serum Metabolic Profiles

**Figure 5:**
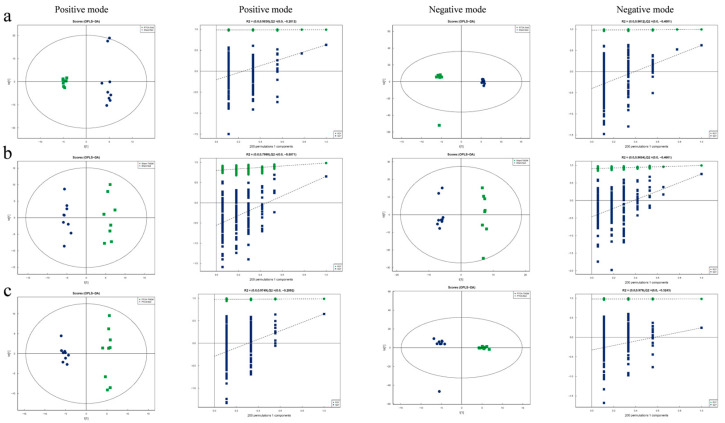
The metabolomic analysis in the exercised PTOA animals. The OPLS-DA scatter plots and validation of the OPLS-DA model via a permutation test in positive mode and negative mode: (**a**) PTOA/Sed versus Sham/Sed, (**b**) Sham/TW2M versus Sham/Sed, and (**c**) PTOA/TW2M versus PTOA/Sed.

As the microbe–host bridge, many metabolites of the gut microbiome could influence host physiology by entering the bloodstream. Therefore, the serum samples from Sham/Sed, Sham/TW2M, PTOA/Sed, and PTOA/TW2M groups were analyzed to explore the serum metabolic profiling using LC–MS. In total, 198 and 143 peak features were identified in positive and negative ion modes, respectively. Subsequently, OPLS-DA was conducted to optimize the separation and identification of metabolites. The validation plots were obtained using 200 permutation tests. The OPLS-DA score plot revealed a distinct separation between the Sham/Sed and PTOA/Sed groups in positive mode (R^2^ = 0.9838, Q^2^ = −0.2012) and negative mode (R^2^ = 0.9812, Q^2^ = −0.4001). R^2^ indicates the goodness of the fit, while Q^2^ reflects the prediction ability of the model. The models effectively divide the samples into two categories, indicating strong reliability and predictive capability (Figure 5a).

**Figure 6:**
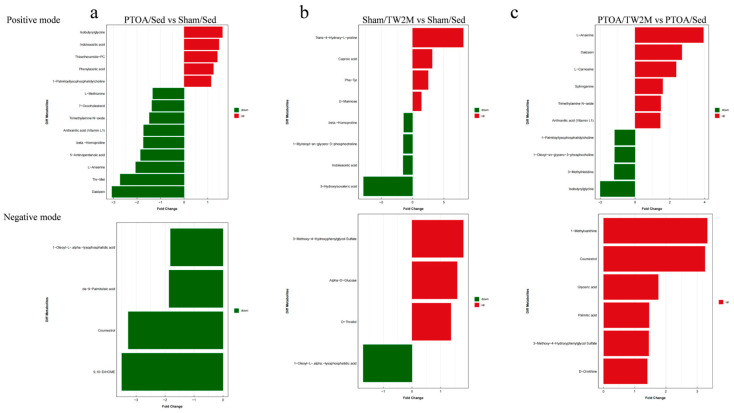
The changed metabolites obtained from serum in the exercised PTOA animals. Histogram of significantly changed metabolites among different groups in positive mode and negative mode: (**a**) PTOA/Sed versus Sham/Sed, (**b**) Sham/TW2M versus Sham/Sed, and (**c**) PTOA/TW2M versus PTOA/Sed.

According to score plots (Figure 5b), there was a noticeable separation trend between the Sham/TW2M and Sham/Sed groups in both positive ion mode (R^2^ = 0.7998, Q^2^ = −0.5571) and negative ion mode (R^2^ = 0.9054, Q^2^ = −0.4661). Moreover, in both positive (R^2^ = 0.9749, Q^2^ = −0.2892) and negative ion modes (R^2^ = 0.979, Q^2^ = −0.3243), clear distinctions between PTOA/TW2M and PTOA/Sed groups (Figure 5c) were observed, indicating that joint injury and exercise cause changes in metabolites. To determine the variables responsible for this separation, the VIP parameter was employed. From the OPLS-DA model, VIP indicated which metabolites played a crucial role in differentiating rat samples. Metabolites with VIP score >1 were selected as potential candidates. Significant differences in variables were identified with *p* value threshold of 0.05. The distinguished different metabolites are displayed in Table 1 and Table 2. In addition, potential metabolites are shown in a histogram according to the fold change parameter. When compared with the Sham/Sed group, five metabolites were up-regulated and nine metabolites were down-regulated in positive mode, as well as four metabolites were down-regulated in the PTOA/Sed group (Figure 6a). Furthermore, compared to the Sham/Sed group, the Sham/TW2M group exhibited four up-regulated and four down-regulated metabolites in positive mode, as well as three up-regulated and one down-regulated metabolites in negative mode (Figure 6b). Interestingly, compared to the PTOA/Sed group, the PTOA/TW2M group showed an up-regulation of six metabolites and a down-regulation of four metabolites in positive mode, with six metabolites up-regulated in negative mode (Figure 6c). It was also observed that joint injury and exercise cause changes in serum metabolic profiles among the four groups.

### 3.4. Exercise-Induced Changes in Serum Metabolism Are Related to the Integrity of Cartilage–Subchondral Bone Unit and Gut Microbiome

Given that the effects of exercise on articular cartilage, subchondral bone unit, gut microbiome, and serum metabolites were defined, respectively, we further investigate the relationship between these impacts through correlation analysis.

**Figure 7:**
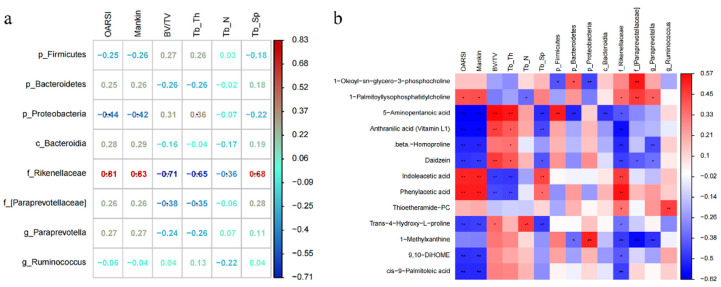
Exercise-induced changes in serum metabolism are related to the integrity of the cartilage–subchondral bone unit and gut microbiome. (**a**) Heat map of Spearman correlations between the abundance of 8 PTOA-relevant gut microbiome and OARSI and Mankin score, as well as subchondral bone micro-CT parameters. The color bar with numbers indicates the correlation coefficient. * *p* < 0.05, ** *p* < 0.01. (**b**) Heat map of Spearman correlations between 13 metabolites with important functions and significant differences and OARSI and Mankin score and subchondral bone micro-CT parameters, as well as 8 PTOA-relevant gut microbiome. * *p* < 0.05, ** *p* < 0.01.

As depicted in Figure 7a, there are identified correlations between joint structural features, such as OARSI score and micro-CT data, and PTOA-relevant microorganisms, including phylum *Firmicutes*, phylum *Bacteroidetes*, phylum *Proteobacteria*, class *Bacteroidia*, family *Rikenellaceae*, family [*Paraprevotellaceae*] and genus *Paraprevotella*, and genus *Ruminococcus*. Interestingly, family *Rikenellaceae* significantly positively correlated with OARSI score (r = 0.81, *p* < 0.01), Mankin score (r = 0.83, *p* < 0.01), and Tb.Sp (r = 0.68, *p* < 0.01) and significantly negatively correlated with BV/TV (r = −0.71, *p* < 0.01) and Tb.Th (r = −0.65, *p* < 0.01), indicating its potential participation in PTOA-relevant cartilage modifications. However, phylum Proteobacteria exhibited stronger negative correlations with OARSI, Mankin, and subchondral bone loss compared to other PTOA-relevant microorganisms. These findings imply a robust connection between structural phenotypes and microorganisms associated with PTOA, where the exercise-responsive gut microbiome family Rikenellaceae may partially contribute to the effects of exercise on cartilage-subchondral bone unit.

Moreover, as shown in Figure 7b, significant correlations were observed between many altered metabolites, OARSI score, Mankin score, micro-CT data, and the relative abundance of PTOA-relevant microbiome. (r > 0.45, *p* < 0.05). For example, 5-Aminopentanoic acid, Anthranilic acid (Vitamin L1), beta-Homoproline, Daidzein, Trans-4-Hydroxy-L-proline, 9,10-DiHOME, and Cis-9-Palmitoleic acid significantly negatively correlated with OARSI and Mankin score, whereas Indoleacetic acid and Phenylacetic acid significantly positively correlated with OARSI and Mankin score. In addition, 5-Aminopentanoic acid, Anthranilic acid (Vitamin L1), and Daidzein had significant positive correlations with BV/TV and Tb.Th, whereas Indoleacetic acid and Phenylacetic acid significantly negatively correlated with BV/TV and Tb.Th. Moreover, 1-Oleoyl-sn-glycero-3-phosphocholine and 1-Palmitoyllysophosphatidylcholine positively correlated with the family [*Paraprevotellaceae*]. 5-Aminopentanoic acid positively correlated with phylum Firmicutes and negatively correlated with phylum Bacteroidetes and class *Bacteroidia*. Anthranilic acid (Vitamin L1), beta-Homoproline, Daidzein, cis-9-Palmitoleic acid, and 1-Methylxanthine were negatively correlated with family *Rikenellaceae*, whereas Indoleacetic acid and Phenylacetic acid were positively correlated with family *Rikenellaceae*. Thioetheramide−PC positively correlated with genus *Ruminococcus*. 1-Methylxanthine positively correlated with phylum *Proteobacteria* and negatively correlated with family [*Paraprevotellaceae*] and genus *Paraprevotella*. These findings indicated that exercise might reduce the relative abundance of family *Rikenellaceae*, which resulted in an increase in anti-inflammatory metabolites and a decrease in inflammatory metabolites, as well as subsequent structural changes in PTOA rats.

## 4. Discussion

In this study, we revealed a possible mechanism of exercise and OA development by the modulation of gut microbiome and metabolites. Our study demonstrates that exercise significantly alleviates cartilage degeneration and subchondral bone loss, potentially through regulating an imbalanced gut microbiome and disturbed serum metabolome (Figure S1). Despite several animal experiments that have demonstrated the therapeutic role of exercise in preventing joint inflammation and cartilage degradation [21,25], the underlying regulatory mechanisms focused on gut microbiome and metabolites are still not well understood.

To delve deeper into this, we employed 16S rRNA gene sequencing techniques to analyze the composition and diversity of gut microbiota in response to joint injury and exercise across different groups. It has been shown that Firmicutes and *Bacteroidetes* are dominant phyla of gut microbiota. In our study, compared with the Sham/Sed group, PTOA/Sed group rats displayed a significant decrease in Firmicutes and an increase in *Bacteroidetes*, while exercise reversed the aberrant gut microbiota composition. Our findings are corroborated by Jiang et al., who found that collagen-induced arthritis rats exhibited a marked reduction in the relative abundance of Firmicutes and an increase in *Bacteroidetes* abundance [33]. Moreover, the decreased *Firmicutes/Bacteroidetes* ratio is relevant to mild inflammatory reaction in high-fat diet rats [34]. Therefore, in our study, the increased *Firmicutes/Bacteroidetes* ratio may interpret the positive anti-inflammatory effect of exercise in OA. As a common intestinal Gram-negative bacterium, the family *Rikenellaceae* was thought to be involved in bone metabolism, with a negative impact on bone resorption and bone density [35]. Several studies have found that an abundance of *Rikenellaceae* may lead to the secretion of proinflammatory cytokines [36]. Prinz et al. have shown that the presence of *Rikenellaceae* is associated with worse OA outcomes, which is consistent with our findings [37]. Our investigation demonstrated that the relative abundance of *Rikenellaceae* was upregulated in PTOA rats and positively correlated with the severity of PTOA-related structural changes and actively responded to exercise treatment. Moreover, the PTOA/Sed group showed an increase in the abundance of genus *Paraprevotella*, which was suppressed by exercise in the PTOA/TW2M group. Our results are supported by Tand et al., who observed that *Paraprevotella* was abundant in the gut microbiota of individuals with depression and associated with OA severity [38]. This finding provides evidence that the protective effect of exercise on OA may be realized by mitigating the presence of pathogenic bacteria, particularly *Rikenellaceae* and *Paraprevotella*, in PTOA rats.

Furthermore, non-targeted LC–MS metabolomics analysis was employed to explore the effects of exercise on serum metabolite profiles of PTOA rats. The results showed that metabolites including Daidzein, Anthranilic acid, and L-Anserine were downregulated, and Indoleacetic acid, Phenylacetic acid, and 1-Palmitoyllysophosphatidylcholine were upregulated in PTOA/Sed group compared to Sham/Sed group. These metabolites are involved in tryptophan metabolism, histidine metabolism, beta-alanine metabolism, phenylalanine metabolism, and amino acid biosynthesis. Additionally, Anthranilic acid and Daidzein were negatively correlated with OARSI score and family *Rikenellaceae*, and they responded actively to exercise treatment.

Anthranilic acid has been found to have potential for anti-inflammatory and anti-cancer activity, and it serves as a crucial agent participating in tryptophan metabolism [39]. There is increasing evidence that has shown that altered tryptophan metabolism has been associated with several disorders, such as erosive hand osteoarthritis and rheumatoid arthritis [40,41]. Daidzein has been reported for effective prevention and treatment of diseases because of its outstanding antioxidant and anti-inflammatory roles [42]. Interestingly, a study [43] indicated that daidzein protected against monosodium iodoacetate-induced OA via reducing the levels of TNF-α, IL-1β, and MMP13 in serum and joint tissue samples and improving cartilage surface fibrillation, which is in line with our discoveries. Our data support that the beneficial effect of exercise in blocking OA pathological progress may be achieved by upregulating the serum level of Anthranilic acid and Daidzein.

Phenyllactic acid is involved in phenylalanine metabolism, which is elevated in OA synovial fluid [44]. Previous studies have shown that increased phenylalanine concentration may be linked to the progression of OA and considered as a pivotal indicator in detecting OA [45,46]. Our results are in agreement with the evidence presented in this study. 1-Palmitoyllysophosphatidylcholine is one of the most prominent lysophosphatidylcholine species, which promotes endothelial pro-inflammatory and pro-adhesive chemokine IL-8 synthesis [47]. The study reported that lysophosphatidylcholine overproduction was positively associated with circulating TNF-α and IL-1β levels [48]. We observed that the level of 1-Palmitoyllysophosphatidylcholine was higher in the PTOA/Sed group compared to the Sham/Sed group. This is consistent with Zhang et al.’s report, in which the concentration of lysophosphatidylcholine was increased in patients with knee OA [49]. The elevated level of 1-Palmitoyllysophosphatidylcholine was decreased as a response to exercise, implying the beneficial anti-inflammatory effect of exercise on OA. These findings provide evidence that the modifications in metabolites are strongly associated with gut microbiota and joint degeneration.

## 5. Limitations

Although our study has elucidated how exercise, through the modulation of gut microbiota and serum metabolites, acts in the context of OA, several limitations should be acknowledged. First, 16S rRNA sequencing technology utilized in our study is predominantly restricted to the taxonomic identification of bacteria, which is incapable of identifying specific microbial species and strains. Further research can employ a whole-genome shotgun metagenomic sequencing approach to generate robust estimates and detailed functional annotations of microbial communities to improve the gut microbiota–joint axis in OA. Second, our sample size was comparatively small, and the findings require further validation in a larger sample size study to strengthen the robustness of the conclusions. Meanwhile, the lack of cross-sectional studies limits the ability to establish causal connections between gut microbiome dysbiosis and OA progression. The direct relationship between joint degeneration and the identified gut microbiota, specifically Firmicutes and *Rikenellaceae*, as well as the putative key metabolites, Daidzein and Anthranilic acid, requires additional experimental verification. In addition, some potential unmeasured confounders, including circadian rhythm and stress levels, were not well controlled, which may influence gut microbiota and metabolite profiles. Moreover, there is a significant disparity in microbiota composition between humans and animals, as evidenced by the fact that 85% of the bacterial genera found in the animal gastrointestinal tract are absent in the human gut [50]. The genetic background of different animal models also affects microbiota composition. These factors present challenges in the translational relevance of animal models for human applications and indicate that an exact correspondence cannot be achieved between animal and human models, or even among different animal models. Finally, PTOA, metabolic syndrome-associated OA, and age-related OA have distinct causes, mechanisms, and clinical presentations, which may lead to varied responses to exercise interventions. Our study exclusively focuses on the effects of exercise on PTOA due to limitations in sample size and the heterogeneity of the animal model.

## 6. Conclusions and Future Perspectives

In summary, our study demonstrates that exercise can serve as a therapeutic agent to mitigate OA through slowing down articular cartilage degeneration and subchondral bone loss, modulating gut microbiota composition, and increasing beneficial metabolites. Moreover, our study highlights that the benefits of exercise on joints might be related to the identified gut microbiota, particularly *Firmicutes* and *Rikenellaceae*, along with the possible key metabolites, Daidzein and Anthranilic acid. The recognized gut microbiota and essential metabolites may be a potential target for exercise intervention, offering clinical potential implications for OA therapy. In addition, these findings provide fresh insights into uncovering the mechanism of exercise treatment for OA.

Furthermore, preclinical animal models only partially mimic the complexity of the human microbiome; there is a need for more high-quality clinical studies or large sample size cross-sectional studies to identify the causal links between gut microbiota and OA progression in future research. Collectively, the innovative concept along with advanced research provides novel direction to develop a therapeutic approach for OA by targeting key metabolites or gut microbiota.

## Figures and Tables

**Table 1:** Significantly different metabolites among the four groups based on OPLS-DA in positive ion mode.

Comparative Group	Metabolites	VIP	Fold Change	*p*-Value
PTOA/Sed versus Sham/Sed	beta-Homoproline	2.400925861	0.576463827	0.001
	Daidzein	1.939621477	0.32599906	0.001
	Phenylacetic acid	1.691965753	1.253189416	0.002
	5-Aminopentanoic acid	1.515618194	0.539227609	0.003
	Anthranilic acid (Vitamin L1)	6.114384325	0.579288897	0.003
	L-Anserine	3.448507904	0.485224903	0.019
	1-Palmitoyllysophosphatidylcholine	3.241282054	1.150208786	0.026
	Trimethylamine N-oxide	2.238067382	0.674405948	0.036
	L-Methionine	1.426522831	0.747339917	0.036
	7-Oxocholesterol	1.109913427	0.727777146	0.040
	Thioetheramide-PC	16.14239843	1.422210189	0.041
	Isobutyrylglycine	1.429748753	1.632478727	0.043
	Indoleacetic acid	1.64116589	1.486730841	0.044
	Thr-Met	1.198055767	0.367348409	0.046
Sham/TW2M versus Sham/Sed	Caproic acid	2.322510891	3.147297216	0.001
	Trans-4-Hydroxy-L-proline	1.722552698	8.129422426	0.001
	3-Hydroxyisovaleric acid	1.1778371	0.127366318	0.001
	1-Myristoyl-sn-glycero-3-phosphocholine	5.221306055	0.673725935	0.005
	Phe-Tyr	1.071113318	2.505113841	0.005
	D-Mannose	1.552197047	1.396176604	0.018
	Indoleacetic acid	1.155273074	0.661480492	0.027
	beta-Homoproline	1.780179794	0.699808117	0.035
PTOA/TW2M versus PTOA/Sed	Daidzein	1.567454134	2.695336031	0.001
	L-Carnosine	1.372057913	2.361096252	0.003
	L-Anserine	6.373354859	3.939618039	0.005
	Isobutyrylglycine	1.578019604	0.501190958	0.012
	Sphinganine	1.018051966	1.602814019	0.013
	Trimethylamine N-oxide	1.98858132	1.48755549	0.015
	Anthranilic acid (Vitamin L1)	3.914777318	1.449357496	0.016
	1-Oleoyl-sn-glycero-3-phosphocholine	12.9633525	0.851623797	0.016
	3-Methylhistidine	1.58895438	0.834093177	0.017
	1-Palmitoyllysophosphatidylcholine	3.387267701	0.852708007	0.033

**Table 2:** Significantly different metabolites among the four groups based on OPLS-DA in negative ion mode.

Comparative Group	Metabolites	VIP	Fold Change	*p*-Value
PTOA/Sed versus Sham/Sed	1-Oleoyl-L-.alpha.-lysophosphatidic acid	2.278466783	0.55020154	0.001
	Coumestrol	2.704798442	0.304959452	0.002
	cis-9-Palmitoleic acid	6.569765019	0.535145129	0.018
	9,10-DiHOME	1.610698643	0.28558991	0.028
Sham/TW2M versus Sham/Sed	3-Methoxy-4-hydroxyphenylglycol sulfate	1.561900233	1.795819177	0.004
	1-Oleoyl-L-.alpha.-lysophosphatidic acid	2.087100203	0.585488284	0.009
	Alpha-D-glucose	1.062456392	1.580477716	0.012
	D-Threitol	1.560157863	1.361687968	0.042
PTOA/TW2M versus PTOA/Sed	Coumestrol	2.781664503	3.25340615	0.002
	Glyceric acid	1.515536125	1.756441364	0.005
	D-Ornithine	2.626868222	1.404355261	0.023
	Palmitic acid	16.40253911	1.458633594	0.024
	1-Methylxanthine	1.558013091	3.327696176	0.029
	3-Methoxy-4-hydroxyphenylglycol sulfate	1.011534776	1.446836056	0.036

## Data Availability

The data presented in this study are available on request from the corresponding author. The raw data were deposited in the NCBI Sequence Read Archive (SRA) (accession numbers for NCBI: BioProject: PRJNA1240216 for 16S rRNA sequencing).

## Supplementary Materials

The following supporting information can be downloaded at: https://www.mdpi.com/article/10.3390/metabo15050341/s1, Figure S1: Schematic diagram of the potential protective mechanism and effects of exercise in the development of osteoarthritis by altering the compositions of gut microbiome and serum metabolites. Exercise can decrease the abundance of family *Rikenellaceae*, increases the production of Anthranilic acid and Daidzein, and prevent cartilage degeneration and the loss of subchondral bone of osteoarthritis joint.

## Abbreviations

OA, osteoarthritis; PTOA, post-traumatic osteoarthritis; SD, Sprague-Dawley; Sham/Sed, Sham/sedentary; Sham/TW2M, Sham/treadmill walking 2 months; PTOA/Sed, PTOA/sedentary; PTOA/TW2M, PTOA/treadmill walking 2 months; DMM, destabilization of the medial meniscus; ACLT, transection of the anterior cruciate ligament; LPS, lipopolysaccharide; PGN, peptidoglycan; FFAs, free fatty acids; SPF, specific pathogen-free; BV/TV, bone volume/tissue volume fraction; Tb.N, trabecular number; Tb.Sp, trabecular separation; Tb.Th, trabecular thickness; EDTA, ethylenediaminetetraacetic acid; MTP, medial tibial plateau; OARSI, Osteoarthritis Research Society International; ELISA, enzyme-linked immunosorbent assay; OTUs, operational taxonomic units; Chao1, Chao richness estimator; QC, quality control; LC–MS, liquid chromatography–mass spectrometry; OPLS-DA, orthogonal partial least-squares discriminant analysis; VIP, variable importance for projection; PCoA, principal coordinate analysis.
