# Supplementary material for: Effects of Exercise on Gut Microbiome and Serum Metabolomics in Post-Traumatic Osteoarthritis Rats
Source: Metabolites. 2025 May 20;15(5):341. doi: 10.3390/metabo15050341 (PMC12113318; doi:10.3390/metabo15050341)
Supplement: Supplementary file 1 [file metabolites-15-00341-s001.zip › metabolites-3595341-supplementary.pdf]

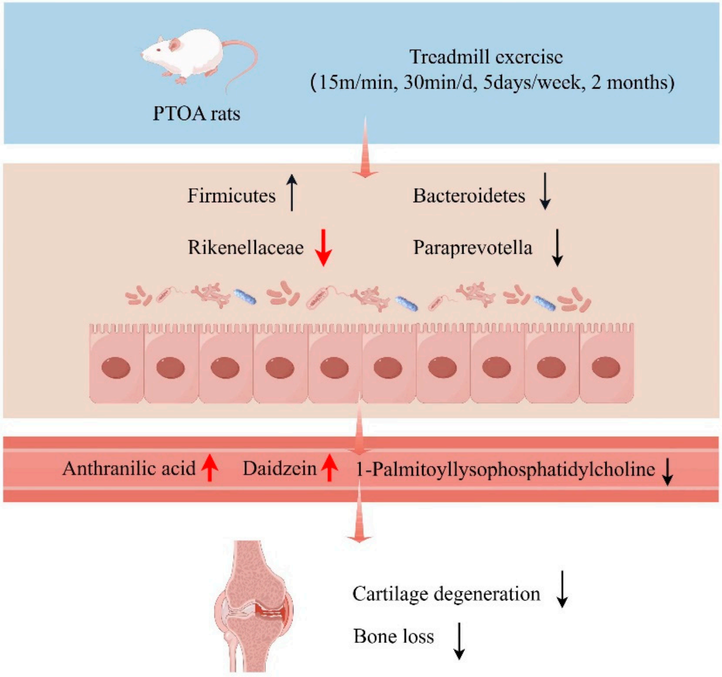

Figure S1. Schematic diagram of the potential protective mechanism and effects of exercise in the development of osteoarthritis by altering the compositions of gut microbiome and serum metabolites. Exercise can decrease the abundance of family Rikenellaceae, increases the production of Anthranilic acid and Daidzein, and prevent cartilage degeneration and the loss of subchondral bone of osteoarthritis joint.
